# Association between Controlling Nutritional Status (CONUT) Score and Body Composition, Inflammation and Frailty in Hospitalized Elderly Patients

**DOI:** 10.3390/nu16050576

**Published:** 2024-02-20

**Authors:** Aurelio Lo Buglio, Francesco Bellanti, Daniela Francesca Pia Carmignano, Gaetano Serviddio, Gianluigi Vendemiale

**Affiliations:** Department of Medical and Surgical Sciences, University of Foggia, Viale Pinto 1, 71122 Foggia, Italy; francesco.bellanti@unifg.it (F.B.); daniela.carmignano@gmail.com (D.F.P.C.); gaetano.serviddio@unifg.it (G.S.); gianluigi.vendemiale@unifg.it (G.V.)

**Keywords:** malnutrition, elderly, hospitalized elderly, screening tools, CONUT score, inflammation, frailty, body composition

## Abstract

The Controlling Nutritional Status (CONUT) score has demonstrated its ability to identify patients with poor nutritional status and predict various clinical outcomes. Our objective was to assess the association between the CONUT score, inflammatory status, and body composition, as well as its ability to identify patients at risk of frailty in hospitalized elderly patients. Methods: a total of 361 patients were retrospectively recruited and divided into three groups based on the CONUT score. Results: patients with a score ≥5 exhibited significantly higher levels of inflammatory markers, such as erythrocyte sedimentation rate (ESR), C-reactive protein (CRP), Neutrophil/Lymphocytes ratio (NLR), main platelet volume (MPV), and ferritin, compared to those with a lower score. Furthermore, these patients showed unfavorable changes in body composition, including a lower percentage of skeletal muscle mass (MM) and fat-free mass (FFM) and a higher percentage of fatty mass (FM). A positive correlation was found between the CONUT score and inflammatory markers, Geriatric Depression Scale Short Form (GDS-SF), and FM. Conversely, the Mini Nutritional Assessment (MNA), Mini-Mental Status Examination, activity daily living (ADL), instrumental activity daily living (IADL), Barthel index, FFM, and MM showed a negative correlation. Frailty was highly prevalent among patients with a higher CONUT score. The receiver operating characteristic (ROC) curve demonstrated high accuracy in identifying frail patients (sensitivity). Conclusions: a high CONUT score is associated with a pro-inflammatory status as well as with unfavorable body composition. Additionally, it is a good tool to identify frailty among hospitalized elderly patients.

## 1. Introduction

Malnutrition, especially among hospitalized older individuals, presents a multifaceted challenge with profound implications for health outcomes [1]. Beyond its conventional association with weight loss, malnutrition is linked to heightened risks, including nosocomial infections, prolonged hospital stays, disability, and, notably, mortality [2,3,4,5]. This intricate scenario is further complicated by inflammaging, a pro-inflammatory status associated with aging and shared in numerous pathological states [6,7]. The altered nutritional status is also associated with increased oxidative stress, characterized by high serum levels of hydroxynonenal (HNE) and malondialdehyde (MDA) protein adducts [8,9]. These protein adducts are involved in several diseases, including metabolic diseases, neurodegenerative diseases, and cancer. HNE and MDA can interact with proteins and enzymes, inducing structural alterations that can lead to functional modification and inactivation [10,11]. These effects can be reflected in DNA damage, cell proliferation, differentiation, and apoptosis [12]. On the other hand, oxidative stress and inflammation can affect nutritional status, leading to anorexia, reduced food intake, and muscle catabolism [13,14]. This interplay between malnutrition, oxidative stress, and inflammaging further contributes to the loss of muscle mass and affects muscle architecture, leading to an increased risk of sarcopenia and frailty [15,16,17]. Frailty tends to increase in prevalence with age and is strongly associated with worse clinical outcomes [18,19,20].

Various validated screening tools for malnutrition exist within the hospitalized elderly population [21]. While these tools exhibit varying degrees of agreement in identifying malnutrition, they consistently demonstrate associations with inflammation, alterations in body composition, and predictive capabilities for diverse clinical outcomes [22,23]. Some evidence has demonstrated the capability of nutritional screening tools to identify frail patients, although with varying accuracy [24,25]. The ability to identify hospitalized patients at higher risk of developing unfavorable clinical outcomes is a goal of paramount importance, aiming to implement preventive clinical measures and personalized clinical approaches [26,27]. Malnutrition, the proinflammatory state, and body composition are among the key factors that have been shown to influence clinical outcomes in hospitalized elderly patients [28,29,30]. The possibility of using a simple and rapid nutritional score capable of predicting alterations in body composition and inflammatory status could prove to be of great clinical utility.

The Controlling Nutritional Status (CONUT) score, a cost-effective and easily administered screening tool, displays notable efficacy in identifying disturbances in nutritional status and predicting various clinical outcomes in different hospitalized populations [5,28,31]. However, there is limited literature regarding its association with inflammatory status and body composition in the hospitalized elderly population. This study aims to explore the association between the CONUT score and nutritional status, body changes, and inflammation in a hospitalized older population. Also, we assess the ability of the CONUT score to identify frail patients.

## 2. Materials and Methods

### 2.1. Study Population and Design

This is a retrospective single-center study conducted at the Department of “Ageing and Internal Medicine”, “Policlinico Riuniti” in Foggia (Italy). Clinical data of 361 patients admitted to our department between September 2022 and March 2023 aged 65 years or older were analyzed. The exclusion criteria were active cancer and acute infection or sepsis. Patients were divided into three groups based on CONUT scores: Normal (0–1), Mild (2–4), and Moderate/Severe (≥5) [32]. The research received approval from our Institutional Review Board at the Policlinico Foggia and was conducted in adherence to the Declaration of Helsinki.

### 2.2. Biochemical Analysis and Data Collection

A blood sample is taken at the time of admission to the hospital for evaluation of hemoglobin, total number of white blood cells (WBC), neutrophils, lymphocytes, platelet, glucose, albumin, creatinine, total cholesterol, high-density lipoproteins (HDL), low-density lipoproteins (LDL), triglycerides, ferritin, main platelet volume (MPV), C-reactive protein (CRP), and erythrocyte sedimentation rate (ESR). Circulant level of pro-inflammatory cytokines such as IL6, Tumor Necrosis Factor α (TNF α), IL4, IL5, Epidermal Growth Factor (EGF), IL8, IL10, and Vascular Endothelial Growth Factor (VEGF), Interferon-γ (IFN γ) were analyzed employing the EV 3513 cytokine biochip array (CV’s < 10%) and competitive chemiluminescence immunoassays (Randox Laboratories Ltd., based in Crumlin, Dublin, UK). The Neutrophil/Lymphocytes ratio (NLR) was calculated by dividing the number of neutrophils by the number of lymphocytes.

### 2.3. Multidimensional Assessment

#### 2.3.1. Nutritional Status and CONUT Score

The nutritional status was assessed using the Mini Nutritional Assessment (MNA) and the CONUT score. The MNA is a validated tool for evaluating hospitalized elderly patients, consisting of 18 items related to anthropometric, general, dietetic, and subjective assessments. The MNA classifies patients into three categories: malnourished (score < 17), at risk of malnutrition (score 17–23.5), and well-fed (score > 23.5) [33,34]. The CONUT score is a straightforward nutritional screening tool that relies on laboratory parameters, including lymphocyte count, total cholesterol, and serum albumin, as detailed in Table 1 [31].

#### 2.3.2. Cognitive and Functional Status

Cognitive function was evaluated through the use of the Mini-Mental State Examination (MMSE), a validated tool that assesses orientation, memory, attention, the capacity to follow verbal instructions, and written language production, along with visuospatial skills [35].

Functional independence was assessed using the Activities of Daily Living (ADL) scale, the Instrumental Activities of Daily Living (IADL) scale, and the Barthel Index. The ADL scale evaluates fundamental daily activities, such as bathing, dressing, or eating, whereas the IADL scale addresses more complex tasks like using public transportation, handling finances, or shopping. ADL scores range from 0 to 6 points, while IADL scores range from 0 to 8 points [36,37].

The Barthel Index is a widely used tool to assess functional independence in the hospitalized elderly population. This index assigns scores based on the patient’s ability to perform essential daily activities such as personal hygiene and mobility. Scores range from 0 to 100, where a higher score indicates greater independence [38]. Depressive symptoms were assessed using the Geriatric Depression Scale short-form (GDS-SF), a 15-item tool validated for use in the elderly. This scale categorizes individuals into the following groups: No depression (0 to 5 points), mild depression (6 to 9 points), and severe depression (10 to 15 points) [39]. The frailty status was assessed based on Fried’s criteria, which include Weight loss, Exhaustion, Low physical activity, Walk time, and Grip strength. No-frail status was defined in the absence of any of the five criteria, Pre-frail status in the presence of one or two criteria, while patients with three or more criteria were classified as Frail [40].

### 2.4. Anthropometric and Body Composition Assessment

Height, body weight, arm, thigh, waist, and calf circumference were measured according to standardized procedures. The weight of bedridden patients was assessed using a LinkoScale 350 system (LikoAB, Luleå, Sweden). Body mass index (BMI) was derived by dividing the body weight by the square of the height measured in meters. Body mass composition was assessed using the bioimpedentiometer BIA 101-F device (Akern/RJL, Florence, Italy), as previously reported [41].

### 2.5. Statistical Analysis

Data were presented as counts and percentages/interquartile range (IQR) for qualitative variables and as mean ± standard deviation (SD) for quantitative variables. The normal distribution of the samples was assessed using the Kolmogorov-Smirnov test. Group comparisons for continuous variables were conducted using either one-way ANOVA or the Kruskal-Wallis test for parametric or non-parametric distributions, respectively. The Tukey test was employed for post-hoc analysis. Nominal and categorical variables were analyzed using Pearson’s Chi-Squared test. Correlation between variables was investigated through Pearson or Spearman coefficients, depending on the distribution of the variables. The performance of the CONUT score in identifying frail patients was assessed through receiver operating characteristic curve (ROC) analysis. *p* Values < 0.05 were considered statistically significant. Statistical analysis was performed using STATA 14.

## 3. Results

**Figure 1:**
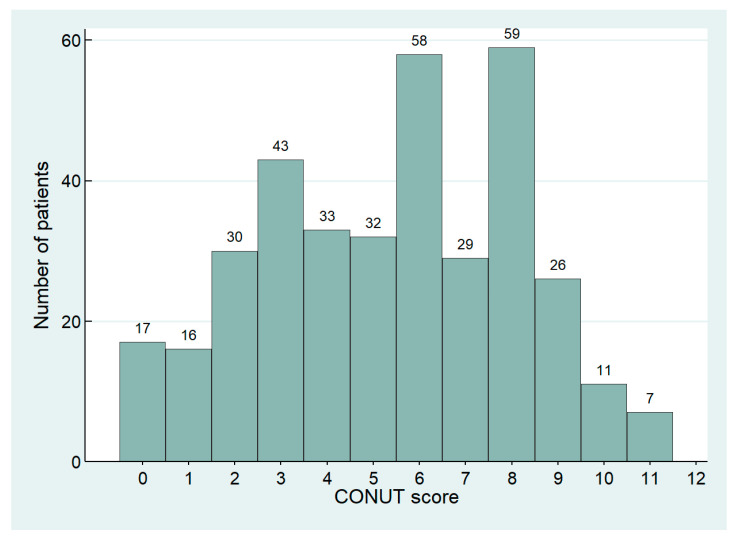
Distribution of CONUT score in the study population.

We retrospectively collected 361 patients, including 215 females, with an average age of 79.2 ± 8.0 years. CONUT distribution in the study population is shown in Figure 1. Patients were divided into the following three groups based on CONUT score: 33 (9.1%) were included in the Normal group, 106 (29.4%) in the Mild group, and 222 (61.5%) in the Moderate/Severe group. Baseline characteristics are summarized in Table 2.

Significant differences were found among groups for age, hemoglobin, neutrophils, lymphocytes, albumin, creatinine, total cholesterol, LDL, and HDL serum values. At post-hoc analysis, patients with moderate/severe malnutrition were significantly older than the other groups as well showed lower haemoglobin, lymphocytes, albumin, total cholesterol, LDL, and HDL values. Neutrophils were higher in patients with a CONUT score of ≥5 compared to another group, while the median value of creatinine was higher in patients with a CONUT score of 0–1 without differences with group Mild. The groups did not differ significantly in genre, number of comorbidities, WBC, platelets, glucose, and triglycerides (Table 2). Hypertension, diabetes, heart failure, ischemic heart chronic disease, chronic obstructive pulmonary disease, and chronic kidney disease, defined as an estimated Glomerular Filtration Rate below 60 mL/min/1.73 m^2^, were the most prevalent comorbidities in all three groups. No differences were found among the groups (Table S1 in Supplementary Materials).

### 3.1. CONUT Score, Multidimensional Evaluation and Prevalence of Frailty

**Figure 2:**
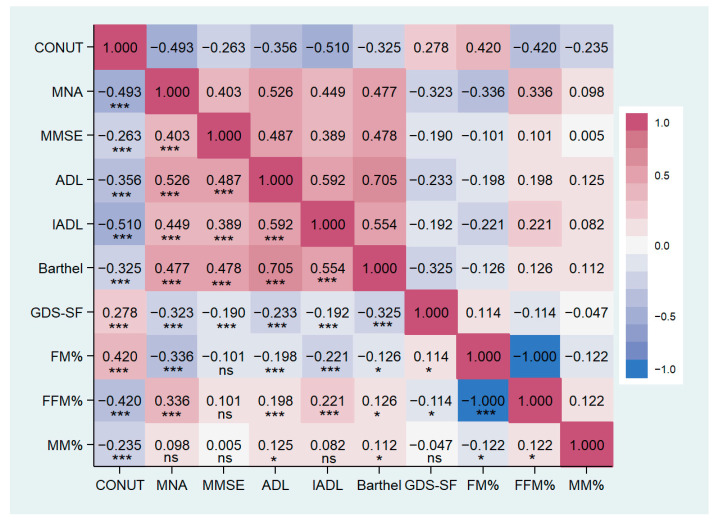
Correlation matrix between CONUT score and clinical characteristics in the study population. CONUT, controlling nutritional status score; MNA, mini nutritional assessment; MMSE, mini-mental state examination; ADL, activity of daily living; IADL, instrumental activity of daily living; GDS-SF, geriatric depression scale short form; FM, fat mass; FFM, free-fat mass; MM, muscle mass. * *p* < 0.05, *** *p* < 0.001, ns: not significant.

Groups significantly differed in nutritional status, cognitive and functional performance, and depressive symptoms (Table 3). Post Hoc analysis showed lower MNA, MMSE, ADL, IADL, and Barthel index in the Moderate/Severe group compared to other groups. GDS score was significantly higher in patients with CONUT score ≥5 than in groups with lower scores. MNA, MMSE, ADL, IADL, and Barthel index are inversely correlated with the CONUT score, while the GDS score shows a positive correlation (details are reported in Table 4 and Figure 2).

**Figure 3:**
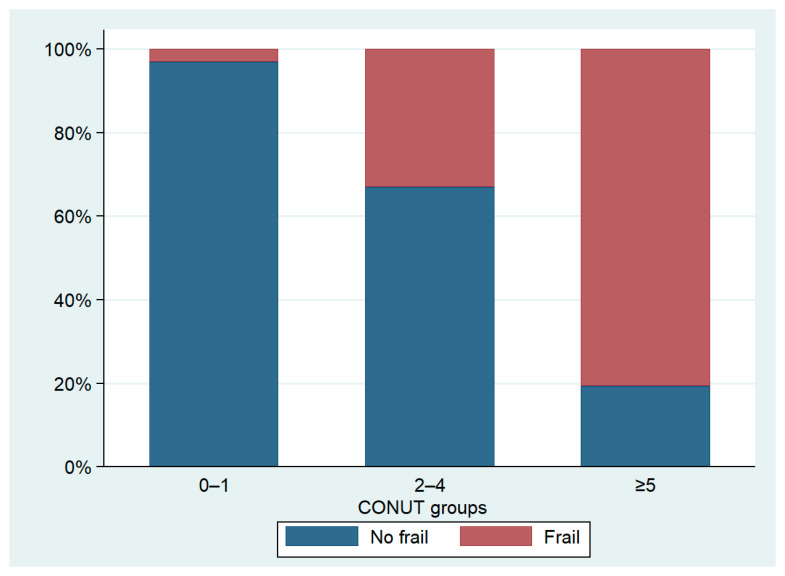
Prevalence of frailty according to CONUT groups.

**Figure 4:**
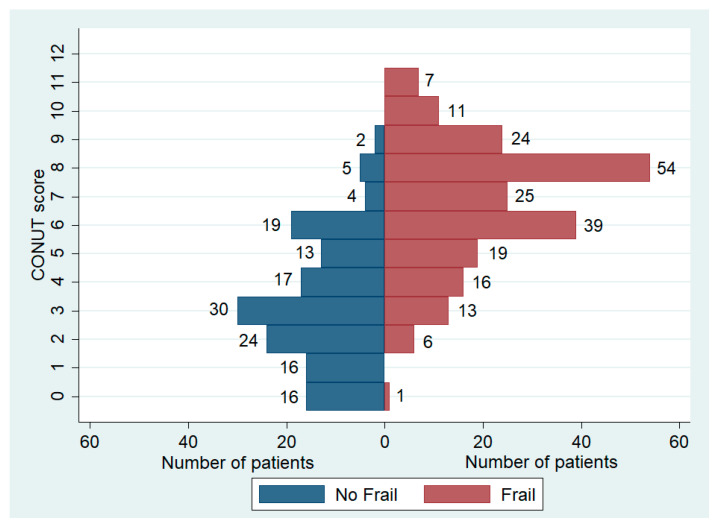
Distribution of CONUT score based on frailty status.

**Figure 5:**
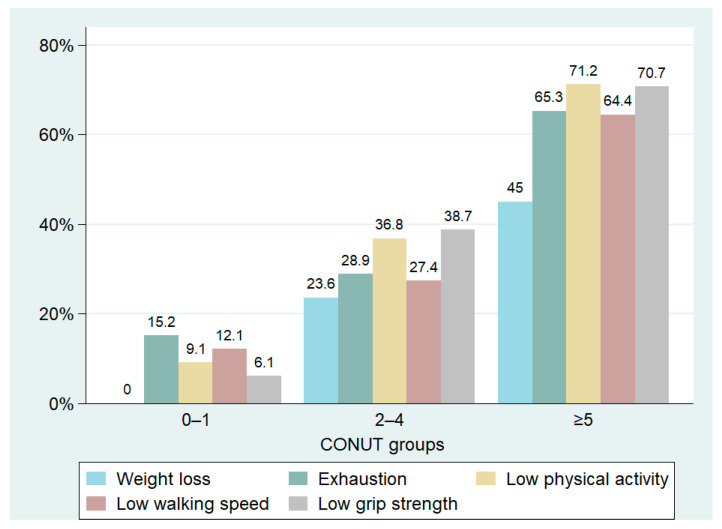
Prevalence of Fried’s criteria based on the CONUT score groups.

We identified 215 frail patients, making up 59.6% of the total population. The prevalence of frailty varied significantly among the three groups, with notably higher cases observed in patients with a CONUT score ≥5. In this subgroup, data showed a prevalence of frail patients reaching up to 80.6%, significantly higher than the other two groups (Table 3, Figure 3). The distribution of CONUT scores based on frailty status is illustrated in Figure 4. Fried’s criteria also exhibited differences among the groups, with a greater prevalence of all criteria observed in the Moderate/Severe group compared to the other groups. Detailed data are provided in Table 3 and Figure 5.

### 3.2. CONUT Score and Inflammatory Markers

**Figure 6:**
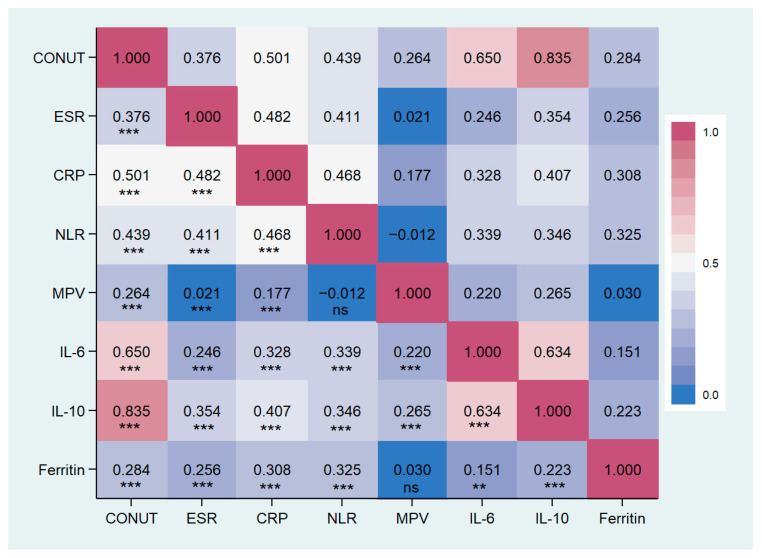
Correlation matrix between CONUT score and inflammatory markers in the study population. CONUT, controlling nutritional status score; ESR, erythrocytes sedimentation rate; CPR, c reactive protein; NLR, Neutrophils/lymphocytes ratio; MPV, main platelet volume; IL, Interleukin. ** *p* < 0.01, *** *p* < 0.001, ns: not significant.

Patients with higher CONUT scores exhibited an increased inflammatory status compared to those with lower scores (see Table 2). ESR, CRP, NLR, MPV, and ferritin levels were significantly elevated in the group with a CONUT score ≥5, as opposed to the other groups. Similarly, within the cytokine profiles, IL-6 and IL-10 levels were notably higher in the moderate/severe malnutrition group than in the other groups, as demonstrated by post hoc analysis. No statistical differences were observed among the remaining inflammatory markers. The correlation analysis showed a significant positive correlation between the CONUT scores and ESR, CRP, NLR, MPV, IL-6, and IL-10. Particularly, the strongest degree of correlation has been found with CRP, IL-6, and IL-10. Data are reported in Table 4 and Figure 6.

### 3.3. CONUT Score, Anthropometric Measures, and Body Composition

BMI, arm circumference, and thigh circumference were significantly lower in patients with moderate/severe malnutrition compared to the other two groups. Patients with a CONUT score ≥5 showed a significantly lower BMI compared to the other groups, as well as a smaller waist circumference than the mild group and a reduced calf circumference compared to the normal group (Table 5). Groups with a CONUT score ≥5 showed an unfavorable body composition compared to the other two groups, characterized by lower muscle mass (MM) and fat-free mass (FFM) associated with a higher percentage of fat mass (FM). No significant differences were found in body water distribution (refer to Table 5). MM and FFM percentages were negatively correlated with CONUT score and positively correlated with FM percentage (Table 4, Figure 2).

### 3.4. CONUT Score as Predictor of Frailty

**Figure 7:**
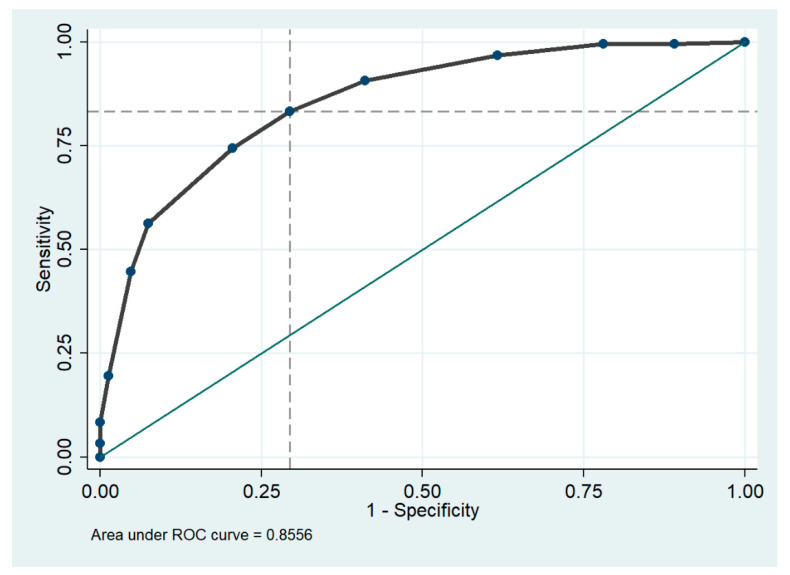
Area under the receiver operating characteristic (ROC) curve for CONUT score cut off in the diagnosis of frailty. The dashed lines on the x and y axes indicate optimal cutoff points for sensitivity and 1-specificity, while the green solid line represents where the test would fall if the results were no better than chance at predicting the presence of a disease.

The Receiver Operating Characteristic (ROC) curve identifies a cut-off value ≥5 for the CONUT score, allowing the identification of frail patients with a sensitivity of 83.3% and a specificity of 70.5%. The Area Under the Curve (AUC) is 0.856 (95% CI 0.817 to 0.894), and Youden’s Index is 0.54. The Positive Predictive Value (PPV) stands at 80.6%, the Negative Predictive Value (NPV) at 74.1%, and the Likelihood Ratio is calculated as 2.83 (Figure 7).

## 4. Discussion

To our knowledge, there are no studies that investigate the association between CONUT score, body composition, and inflammatory status. The principal findings of this study were: (1) a higher CONUT score was associated with elevated levels of inflammatory markers; (2) body composition changes among groups based on the CONUT score, with lower MM and FFM and higher FM in patients with a score ≥5 compared to a lower score; (3) a high CONUT score was associated with a higher prevalence of frailty.

Malnutrition plays a key role in hospitalized elderly patients, affecting various clinical outcomes and emerging as among the most relevant risk factors for prolonged length of stay, mortality, and increased hospitalization costs [42,43]. Poor nutritional status presents a higher prevalence among hospitalized elderly patients and represents one of the most important causes of in-hospital complications [44]. We found a high prevalence of malnutrition in the study population, with more than 90% of the patients exhibiting impaired nutritional status and more than half showing moderate to severe malnutrition according to the CONUT score. Accordingly, previous studies have shown a high prevalence of malnutrition among hospitalized elderly patients [23,45]. In a recent observational and multicenter study, 34.8% of patients over 70 years were malnourished according to GLIM criteria [46]. Skeie E. et al. found a prevalence of patients ‘at risk of malnutrition’ equal to 30.9%, as evaluated with the NRS 2002 score, while Alzharani H et al. identified 29% malnourished patients [47]. No differences in gender among groups were found in our study. The data on the association between malnutrition and gender in hospitalized patients are inconsistent. Some studies report a higher prevalence in males, while others indicate a greater risk of undernutrition in females [3,48]. However, other studies did not report differences in sex-related [5,43]. These different results could be influenced by differences in the study populations. Patients with higher CONUT scores were older compared to other groups. This is consistent with the literature; in fact, old age is among the most significant risk factors for malnutrition, especially among hospitalized patients, due to several factors such as diminished taste or olfactory dysfunction, delayed gastric emptying, or impaired cognitive status [1,23].

The three groups in our study population significantly differ in clinical characteristics. Interestingly, groups stratified by CONUT score demonstrated a well-defined association with nutritional, cognitive, and functional performance. Data across the literature regarding the association between CONUT and MNA scores are lacking and inconsistent. Formiga et al. found a higher ability of MNA to identify patients with abnormal nutritional status compared to CONUT score without a significant correlation between the two scores [49]. Likely, Cabrè et al. observed a weak agreement between the CONUT score and MNA in older people [50]. Conversely, Uemura Y et al. observed a significant correlation between CONUT score and MNA-SF, as well as CONUT score and GNRI [51]; Gonzalez et al. showed a high correlation between CONUT score and Subjective Global Assessment [52]. We found that patients with a score ≥5 showed a lower MNA score compared to the other groups, demonstrating a significant inverse correlation.

Unlike the MNA score, the CONUT score is a simplified tool primarily utilizing laboratory test data. Specifically, it incorporates albumin, cholesterol, and lymphocyte count levels, which are repeatable, independent of the patient’s vigilance status, not operator-dependent, and easily measurable. This simplicity makes CONUT score more feasible in acute hospital settings compared to the comprehensive approach of MNA, which involves interviews, medical history, and physical measurements. Additionally, the laboratory parameters used in the CONUT score are suitable for monitoring during therapy [53].

The group with a score ≥5 exhibited impaired cognitive performance and a higher prevalence of depression symptoms, in line with what has been previously reported [54]. Physical autonomy was also lower in patients with higher CONUT scores. Previously, studies have shown how malnutrition is associated with impaired cognitive and functional status, as well as poor physical performance, particularly in dynamic physical performance, as recently reported [55,56,57]. However, static physical performance is also associated with malnutrition to the extent that the reduction in muscle strength has been observed as a predictor of nutritional status [58]. Cognitive and functional impairment are important risk factors for unfavorable clinical outcomes in hospitalized patients [59,60,61]. Considering the high prevalence of patients with higher CONUT scores presenting with impaired cognitive and physical function, as highlighted in our study population, the CONUT score could be helpful in identifying patients with alterations in these clinical domains and who are at risk for unfavorable clinical outcomes. Also, we found a significantly higher prevalence of depressive symptoms in patients with high CONUT scores compared to other groups. Malnutrition is a recognized risk factor for the development of depression in various populations, including hospitalized patients [62,63,64]. Recently, a significant association between CONUT score and risk of depression was observed in patients hospitalized for acute ischemic stroke [65]. In the correlation analysis, we observed a positive and significant correlation between the CONUT score and MMSE, ADL, IADL, and Barthel index, as well as a positive correlation with the GDS score.

Aging is associated with several unfavorable changes in metabolism and body composition, increasing the risk of malnutrition. Simultaneously, malnutrition is an important risk factor for changes in body composition [66,67,68]. We observed differences in body composition among groups, noting a lower percentage of MM and FFM, along with a significantly higher percentage of FM in patients with a score ≥5 compared to those with a lower score. Notably, patients with a higher score were also older compared to the other groups. Age and poor nutritional status mutually affect body mass composition, facilitating the loss of FFM [66]. Especially in older people, different social, physiological, and pathological conditions could lead to inadequate dietary intake, favoring the onset of malnutrition. A deficiency in nutrient intake or absorption can lead to a decrease in muscle mass, resulting in reduced physical activity and impaired muscle function [69,70]. The impact of malnutrition on body composition is so significant that the reduction of muscle mass is one of the diagnostic criteria for malnutrition in the latest consensus from the global clinical nutrition community (GLIM) [71]. Malnutrition, sarcopenia, and physical performance are interrelated and share numerous risk factors [72,73]. The loss of muscle, reduced muscle strength, and physical performance are the key changes that lead to sarcopenia, and sarcopenia is one of the most important risk factors for frailty [74,75].

The present study demonstrates that a high CONUT score is significantly associated with a higher prevalence of frailty. We observed that more than 80% of frail patients were in the group with moderate/severe malnutrition, while 33% were in the group with mild malnutrition. Moreover, among Fried’s score items, low physical activity and weak grip strength were the most frequently observed criteria in patients with a score ≥2. Nevertheless, in the moderate/severe group, almost all criteria consistently exhibited a high prevalence. Malnutrition is closely related to frailty status. Low nutritional intake, poor diet quality, and insufficient energy intake play a key role in the development of malnutrition, increasing the risk of frailty, especially among elderly patients [76]. Poor nutritional intake adversely affects body composition, resulting in the loss of skeletal muscle mass and an increase in fatty mass. These unfavorable changes contribute to an increased risk of frailty. Conversely, frail individuals were found to be five times more likely to be at risk of malnutrition. Current literature presents frailty and malnutrition as two concepts that share both common and varied pathophysiological mechanisms [77]. Muscle architecture, characterized by muscle thickness, fascicle length, and pennation angle, plays a crucial role in muscle performance and strength, and it is closely linked to nutritional status. Malnutrition is associated with altered muscle architecture, which may explain its impact on muscle performance [17]. One of the other most important shared elements is a pro-inflammatory status. In fact, both malnutrition and frailty are associated with an increase in inflammation indices [14,78]. Our study emphasizes a close association between the CONUT score and inflammatory status. Specifically, ESR, CRP, NLR, MPV, and ferritin levels were higher in patients with malnutrition compared to other groups, also showing a significant positive correlation (Table 4 and Figure 6). Additionally, the circulating levels of IL-6 and IL-10 were significantly elevated in the group with poor nutritional status compared to the other groups. Previous studies have demonstrated a strong association between circulating inflammatory markers, such as CRP, IL-6, IL-10, and TNF-α, and malnutrition. This association has been observed both in general populations and in special groups, such as the elderly or cancer patients [67,79,80,81,82]. Inflammation is an element involved in several pathologies and has been recognized as a pivotal factor contributing to disease-related malnutrition, inducing anorexia, decreased food intake, muscle catabolism, and insulin resistance, ultimately promoting a catabolic state. Recent compelling data indicate that inflammation plays a role in shaping the response to nutritional interventions [14,83,84]. This can justify the association between the CONUT score and inflammatory markers.

Frailty is also associated with pro-inflammatory status and, in particular, CRP and IL-6 [78,85].

In the recently introduced GLIM diagnosis of malnutrition, the presence of markers of inflammation is included among the etiological criteria [86]. Both malnutrition and inflammation affect muscle metabolism, suppressing muscle protein synthesis and activating proteolysis, thus facilitating the development of sarcopenia [87,88]. Frailty is one of the most important syndromes in older people and is associated with several poor clinical outcomes, such as immobility or disability, increased risk of hospitalization, prolonged length of stay, and higher in-hospital mortality [89]. The importance of malnutrition and frailty in the hospital setting has been highlighted in several guidelines, which recommend routine screening for these conditions [90,91]. Previous studies have assessed the effectiveness of nutritional screening tools, such as the MNA-SF or MUST, in identifying frail patients during hospitalization, considering the strong link between frailty and malnutrition [24,76]. The frequent coexistence of frailty and undernutrition in hospitalized older adults emphasizes the practicality of employing a single screening tool for both conditions, streamlining practice for time-constrained healthcare professionals [90,92]. The Receiver Operating Characteristic (ROC) curves demonstrated the high performance of the CONUT score in identifying frail patients, suggesting its potential use as a quick and straightforward screening tool in an acute hospital setting.

However, this study has some limitations. First, this is a single-center study, and the retrospective design may not account for certain confounding factors. Second, the limited sample size limits the statistical power of the study and, in addition to influencing the heterogeneous distribution of sample sizes, may have effects on the statistical analysis. Third, information on statin use, which can impact the CONUT score by influencing cholesterol levels, was not collected from our patients.

Finally, potential biases may exist in this study due to its single-center nature, thereby limiting the generalizability of the results to other populations.

## 5. Conclusions

In conclusion, our study sheds light on the intricate connections between CONUT score, inflammatory status, body composition, and frailty in hospitalized elderly patients. The findings reveal that a high CONUT score is strongly associated with a higher prevalence of frailty, unfavorable body composition changes, and a prof-inflammatory status. This association has significant implications for understanding the pathophysiological pathways linking malnutrition, frailty, and inflammation. Further research with multicenter, large-sample, and prospective clinical studies is warranted to validate and expand upon these findings.

## Figures and Tables

**Table 1:** Controlling Nutritional Status (CONUT) score calculation.

	Nutritional Status			
Variables	Normal	Light	Moderate	Severe
Albumin (g/dL) Score	≥3.5 0	3.0–3.49 2	2.5–2.9 4	<2.5 6
Total lymphocyte (/mm^3^) Score	>1600 0	1200–1599 1	800–1199 2	<800 3
Total cholesterol (mg/dL) Score	>180 0	140–180 1	100–139 2	<100 3
Screening total score	0–1	2–4	5–8	9–12

Abbreviation: CONUT, controlling nutritional status.

**Table 2:** Baseline characteristics of the patients according to Normal (0–1), Mild (2–4), or Moderate/Severe (≥5) CONUT score.

	Normal (Score 0–1) n. 33 (9.1%)	Mild (Score 2–4) n. 106 (29.4%)	Moderate/Severe (Score ≥ 5) n. 222 (61.5%)	*p* Value
Age, years	74.1 ± 7.2	76.9 ± 7.7	81.1 ± 7.8	**<0.001**
Genre F, *n* (%)	19 (57.6)	62 (58.5)	134 (60.4)	0.922
Comorbities, ≥3	12 (36.4)	47 (44.3)	103 (46.4)	0.553
Haemoglobin, g/dL	13.5 ± 1.5	12.1 ± 2.0	10.9 ± 1.8	**<0.001**
WBC, *n*/mm^3^	6900 [5800–9225]	7130 [5350–8790]	7400 [5000–11,090]	0.918
Neutrophils, *n*/mm^3^	4319 [3247–5638]	4613 [3445–6381]	5639 [3368–8940]	**0.021**
Lymphocytes, *n*/mm^3^	1991 [1687–2449]	1473 [1065–2160]	981 [730–1370]	**<0.001**
Platelet, 10^3^/mm^3^	202 [181–242]	193 [136–245]	189 [126–236]	0.249
Glucose, mg/dL	100 [89–120]	107 [88–141]	107 [82–139]	0.598
Albumin, g/dL	4.0 ± 0.4	3.4 ± 0.4	2.8 ± 0.4	**<0.001**
Creatinine, mg/dL	0.85 [0.70–1.07]	0.94 [0.77–1.27]	1.06 [0.80–1.49]	**0.015**
Total cholesterol, mg/dL	185 [172–215]	164 [144–189]	125 [105–147]	**<0.001**
LDL, mg/dL	113 [105–127]	98 [86–126]	78 [60–96]	**<0.001**
HDL, mg/dL	49 [42–62]	45 [34–55]	33 [25–40]	**<0.001**
Triglycerides, mg/dL	131 [77–171]	106 [87–149]	103 [73–142]	0.182
ESR, mm/h	9.0 [4.5–63.0]	30.0 [7.0–76.7]	66.0 [40.0–88.2]	**<0.001**
CRP, ng/mL	2.9 [1.3–4.5]	7.0 [3.7–10.0]	20.9 [12.4–43.7]	**<0.001**
NLR	1.3 [1.2–4.5]	2.2 [1.6–3.5]	5.4 [3.7–7.1]	**<0.001**
MPV, fL	10.1 ± 0.9	11.1 ± 1.2	11.7 ± 1.4	**<0.001**
Ferritin, ng/mL	87 [29–158]	98 [35–207]	182 [57–435]	**<0.001**
IL1α, pg/mL	0.8 [0.4–1.2]	0.6 [0.3–1.0]	0.7 [0.3–1.0]	0.258
IL1β, pg/mL	0.1 [0.0–0.3]	0.7 [0.3–0.9]	0.5 [0.3–0.8]	0.390
IL2, U/mL	0.2 [0.1–0.7]	0.6 [0.3–1.0]	0.4 [0.2–0.6]	0.683
IL6, pg/mL	2.3 [1.0–2.9]	5.1 [2.9–12.6]	18.6 [14.2–19.8]	**<0.001**
IL8, pg	67.5 [39.7–123.4]	104.1 [56.4–125.0]	121.6 [92.8–194.3]	0.754
IL10, pg/mL	0.5 [0.3–0.6]	1.26 [0.9–1.7]	2.2 [1.7–3.0]	**<0.001**
TNFα, pg/mL	3.3 [3.2–3.7]	3.6 [2.3–6.6]	3.7 [2.6–4.9]	0.236
EGF, pg/mL	16.0 [9.7–28.9]	11.4 [7.6–13.2]	8.7 [7.1–13.8]	0.079
VEGF, pg	196.2 [124.3–349.2]	169.0 [128.4–299.3]	220.0 [161.0–295.6]	0.944
INFγ, pg/mL	0.9 [0.3–0.9]	0.9 [0.5–4.8]	0.7 [0.1–0.9]	0.514

Data are expressed as mean (±standard deviation), median [interquartile range], or *n* (percentage) as appropriate. Abbreviation: F, female; WBC, white blood cells; LDL, low-density lipoprotein; HDL, high-density lipoprotein; ESR, erythrocytes sedimentation rate; CPR, c reactive protein; NLR, Neutrophils/lymphocytes ratio; MPV, main platelet volume; TNFα, tumor necrosis factor α; VEGF, vascular endothelial growth factor; EGF, epidermal growth factor; IL, interleukin; INFγ, interferon γ. *p*-value < 0.05 was considered statistically significant (in bold).

**Table 3:** Multidimensional evaluation in patients at baseline, according to study groups.

	Normal (Score 0–1) n. 33 (9.1%)	Mild (Score 2–4) n. 106 (29.4%)	Moderate/Severe (Score ≥ 5) n. 222 (61.5%)	*p* Value
MNA, score	25.1 ± 2.1	21.9 ± 4.2	17.4 ± 4.4	**<0.001**
MMSE, score	23.9 ± 3.8	22.2 ± 7.6	17.9 ± 8.7	**<0.001**
ADL, score	5 [4–6]	6 [5–6]	4 [2–6]	**<0.001**
IADL, score	7 [6–7]	7 [4–8]	3 [1–5]	**<0.001**
Barthel, score	95 [85–95]	90 [60–95]	70 [45–90]	**<0.001**
GDS-SF, score	1 [1–3]	3 [1–6]	5 [3–7]	**<0.001**
Frail, *n* (%)	1 (3.0)	35 (33.0)	179 (80.6)	**<0.001**
Fried’s score items				
Weight loss, *n* (%)	0 (0.0%)	25 (23.6%)	100 (45.0%)	**<0.001**
Exhaustion, *n* (%)	5 (15.2%)	20 (18.9%)	145 (65.3%)	**<0.001**
Low physical activity, *n* (%)	3 (9.1%)	39 (36.8%)	158 (71.2%)	**<0.001**
Low walking speed, *n* (%)	4 (12.1%)	29 (27.4%)	143 (64.4%)	**<0.001**
Low grip strength, *n* (%)	2 (6.1%)	41 (38.7%)	157 (70.7%)	**<0.001**

Data are presented as mean (±standard deviation), median [interquartile range], or *n* (percentage) as appropriate. MNA, mini-nutritional assessment; MMSE, mini-mental state examination; ADL, activity of daily living; IADL, instrumental activity of daily living; GDS-SF, geriatric depression scale short form; *p*-value < 0.05 were deemed statistically significant (in bold).

**Table 4:** Correlation analysis of the CONUT score with laboratory and clinical features.

	*r*	*p* Value
ESR, mm/h	0.376	**<0.001**
CRP, ng/mL	0.501	**<0.001**
NLR	0.439	**<0.001**
MPV, fL	0.264	**<0.001**
IL-6, pg/mL	0.650	**<0.001**
IL-10, pg/mL	0.835	**<0.001**
Ferritin, ng/mL	0.284	**<0.001**
MNA, score	−0.493	**<0.001**
MMSE, score	−0.263	**<0.001**
ADL, score	−0.356	**<0.001**
IADL, score	−0.510	**<0.001**
Barthel, score	−0.325	**<0.001**
GDS-SF, score	0.278	**<0.001**
FM, %	0.420	**<0.001**
FFM, %	−0.420	**<0.001**
MM, %	−0.235	**<0.001**

Abbreviations: ESR, erythrocytes sedimentation rate; CPR, c reactive protein; NLR, Neutrophils/lymphocytes ratio; MPV, main platelet volume; IL, Interleukin; MNA, mini nutritional assessment; MMSE, mini-mental state examination; ADL, activity of daily living; IADL, instrumental activity of daily living; GDS-SF, geriatric depression scale short form; FM, fat mass; FFM, free-fat mass; MM, muscle mass. *p*-value < 0.05 were deemed statistically significant (in bold).

**Table 5:** Anthropometric parameters and body composition in the study population, at baseline.

	Normal (Score 0–1) n. 33 (9.1%)	Mild (Score 2–4) n. 106 (29.4%)	Moderate/Severe (Score ≥ 5) n. 222 (61.5%)	*p* Value
BMI, kg/m^2^	28.0 [24.8–31.4]	27. 0 [24.7–30.9]	25.1 [22.6–29.1]	**<0.001**
Arm circumference, cm	28.9 ± 3.5	27.9 ± 5.2	25.1 ± 4.7	**<0.001**
Thigh circumference, cm	52.9 ± 8.6	44.0 ± 7.5	39.8 ± 7.4	**<0.001**
Waist circumference, cm	101.8 ± 14.3	105.7 ± 17.8	99.6 ± 13.9	**0.003**
Calf circumference, cm	34.5 ± 3.7	31.5 ± 5.5	29.4 ± 5.6	**<0.001**
FM, %	32.4 [26.8–36.5]	34.1 [30.2–43.6]	42.4 [39.2–46.1]	**<0.001**
FFM, %,	67.6 [63.4–73.2]	65.9 [56.4–69.8]	57.6 [53.9–60.8]	**<0.001**
MM, %	34.0 [29.3–39.2]	30.6 [25.5–35.3]	28.3 [24.3–33.3]	**<0.001**
TBW, %	54.5 [50.6–59.3]	51.2 [45.1–56.0]	51.8 [45.5–58.2]	0.188
ECW, %	50.1 [46.2–55.7]	51.4 [46.4–56.7]	51.7 [47.5–58.6]	0.519
ICW, %	49.9 [44.2–53.7]	48.6 [43.3–53.6]	48.2 [41.4–52.5]	0.519

Data are presented as mean (±standard deviation), median [interquartile range], or *n* (percentage) as appropriate. Abbreviations: BMI, body mass index; FM, fat mass; FFM, free-fat mass; MM, muscle mass; TBW, total body water; ECW, extracellular water; ICW, intracellular water. *p*-Value < 0.05 were deemed statistically significant (in bold).

## Data Availability

The data presented in this study are available on request from the corresponding author. The data are not publicly available due to ethical and privacy restrictions.

## Supplementary Materials

The following supporting information can be downloaded at: https://www.mdpi.com/article/10.3390/nu16050576/s1, Table S1. Prevalence of comorbidities on hospital admission according to study groups.
