# Supplementary material for: Association between Controlling Nutritional Status (CONUT) Score and Body Composition, Inflammation and Frailty in Hospitalized Elderly Patients
Source: Nutrients. 2024 Feb 20;16(5):576. doi: 10.3390/nu16050576 (PMC10935156; doi:10.3390/nu16050576)
Supplement: Supplementary file 1 [file nutrients-16-00576-s001.zip › nutrients-2866715-supplementary.pdf]

Supplementary Materials

Table S1. Prevalence of comorbidities on hospital admission according to study groups.

	Normal (Score 0–1) n. 33 (9.1%)	Mild (Score 2–4) n. 106 (29.4%)	Moderate/Severe (Score ≥ 5) n. 222 (61.5%)	*p* Value
Hypertension	26 (78.8%)	68 (64.2%)	137 (61.7%)	0.162
Diabetes	8 (24.2%)	32 (30.2%)	77 (34.7%)	0.413
Heart failure	9 (27.3%)	29 (27.4%)	65 (29.3%)	0.924
Ictus	3 (9.1%)	14 (13.2%)	48 (21.6%)	0.067
IHCD	8 (24.2%)	28 (26.5%)	65 (29.3%)	0.762
Atrial fibrillation	2 (6.1%)	21 (19.8%)	54 (24.3%)	0.052
COPD	11 (33.3%)	49 (46.2%)	96 (43.2%)	0.426
CKD	20 (60.6%)	67 (63.2%)	122 (55.0%)	0.348
Cirrhosis	1 (3.0%)	8 (7.5%)	30 (13.5%)	0.085

Data are expressed as n (percentage). IHCD, ischemic heart chronic disease; COPD, chronic obstructive. pulmonary disease; CKD, chronic kidney disease.
